# Human pregnane X receptor is expressed in breast carcinomas, potential heterodimers formation between hPXR and RXR-alpha

**DOI:** 10.1186/1471-2407-8-174

**Published:** 2008-06-19

**Authors:** Isabel Conde, María VT Lobo, Javier Zamora, Julio Pérez, Francisco J González, Emilio Alba, Benito Fraile, Ricardo Paniagua, María I Arenas

**Affiliations:** 1 Department of Cell Biology and Genetics, University of Alcalá, 28871 Alcalá de Henares, Madrid, Spain; 2 Clinical Biostatistics Unit, Hospital Ramón y Cajal, CIBER Epidemiología y Salud Pública (CIBERESP), 28034 Madrid, Spain; 3 Department of Medical Oncology, Hospital Universitario Virgen de la Victoria, 3091, 29010 Málaga, Spain; 4 Department of Cellular and Molecular Physiopathology, Centro de Investigaciones Biológicas, CSIC, 28040 Madrid, Spain

## Abstract

**Background:**

The human pregnane X receptor (hPXR) is an orphan nuclear receptor that induces transcription of response elements present in steroid-inducible cytochrome P-450 gene promoters. This activation requires the participation of retinoid X receptors (RXRs), needed partners of hPXR to form heterodimers. We have investigated the expression of hPXR and RXRs in normal, premalignant, and malignant breast tissues, in order to determine whether their expression profile in localized infiltrative breast cancer is associated with an increased risk of recurrent disease.

**Methods:**

Breast samples from 99 patients including benign breast diseases, *in situ *and infiltrative carcinomas were processed for immunohistochemistry and Western-blot analysis.

**Results:**

Cancer cells from patients that developed recurrent disease showed a high cytoplasmic location of both hPXR isoforms. Only the infiltrative carcinomas that relapsed before 48 months showed nuclear location of hPXR isoform 2. This location was associated with the nuclear immunoexpression of RXR-alpha.

**Conclusion:**

Breast cancer cells can express both variants 1 and 2 of hPXR. Infiltrative carcinomas that recurred showed a nuclear location of both hPXR and RXR-alpha; therefore, the overexpression and the subcellular location changes of hPXR could be considered as a potential new prognostic indicator.

## Background

The human pregnane X receptor (hPXR, also known as SXR) is a member of the NR1I2 subfamily [1]. This receptor presents different isoforms that are differentially activated by a remarkably diverse collection of compounds including both xenobiotics and natural steroids [2]. PXR orthologs show marked differences in their activation profiles between species; thus, pregnenolone 16α-carbonitrile is an efficacious activator of mouse and rat PXR, but has much less activity on the human and rabbit receptors. Conversely, rifampicin activates the human and rabbit PXR but has no activity on the mouse or rat receptors [3].

PXR is a needed partner of RXRs [4] to form heterodimers that induce transcription from ER6 [5] or IR6 [6] response elements present in steroid-inducible *cytochrome P450 *(*CYP*) gene promoters [7]. *Cytochrome P450 *constitutes a multigene family of hemoproteins responsible for the metabolism of numerous xenobiotics, including therapeutic drugs, environmental chemicals and dietary constituents, as well as endogenous compounds such as steroids and bile acids [8]. Kliewer et al. [3] demonstrated in mice that the strong activation of PXR evoked by the pregnane compounds seemed to be mediated by *CYP3A *induction; this effect also appeared in the homologous counterparts of rat, rabbit, and humans [5,6,9,10].

CYP3A and hPXR are mainly expressed in the liver and the intestine, and, to a lesser extent, in kidney and lung [11]; in addition CYP3A enzymes have been found in human breast cancer tissue [12,13]. The tissue distribution and the relative abundance of hPXR mRNA resemble CYP3A expression very closely, suggesting that hPXR may be important not only for induction but also for constitutive expression of these enzymes [11]. Dotzlaw et al. [14] have shown that the level of hPXR mRNA did not differ between breast tumours and their adjacent matched normal breast tissues; however, among different breast tumour types the expression of hPXR mRNA is diverse. This suggests that hPXR is not significantly altered during tumorigenesis but may display changes related to the cancer phenotype and the degree of differentiation [14]. However, Miki et al. [15] studied samples of atypical ductal hyperplasia, ductal carcinoma *in situ *and invasive ductal carcinoma of the human breast and they detected the presence of neither hPXR mRNA nor protein in non-neoplastic breast tissues suggesting that hPXR is predominantly expressed in carcinoma cells.

Several studies have implicated different cytochrome P450 proteins in the mechanisms of resistance to antiestrogens (tamoxifen and toremifene), taxanes and other anticancer compounds. Therefore, the study of the expression and regulatory pathways of P450 in cancer became an active research field [16,17]; in contrast, studies concerning hPXR are rarely found in the literature. Because hPXR is related to the response to different antitumoural treatments, we have investigated the distribution of this orphan receptor and its needed partner RXRs in normal, premalignant, and malignant breast tissues. Also, we analysed its relationship with the patient's clinicopathological data to elucidate whether some differences in the pattern of expression of these proteins occurred and whether these differences could be valuable for prognostic purposes.

## Methods

### Patients and histological samples

Breast samples from 99 patients randomly selected and diagnosed by the Pathology Service of the Hospital Príncipe de Asturias and Hospital Virgen de la Victoria were used with the consent of the patients and permission of the Ethics Committees of Hospitals. Glandular lesions were classified as follows: 12 cases of benign proliferative diseases (BBDs) including ductal and lobular hyperplasia, apocrine metaplasia, fibroadenoma and fibrocystic changes; 10 carcinomas *in situ *(CIS); 77 infiltrative carcinomas, 54 ductal (IDC) and 23 lobular (ILC). Samples were processed for immunohistochemistry (formalin fixation and paraffin embedding) and for Western blot analysis (frozen with liquid nitrogen).

All infiltrative tumour samples were classified by the TNM system; after surgery, the hormonal status of each tumour was evaluated. These patients (from 35 to 91 years of age) were diagnosed of localized breast cancer between 1998 and 2000 and they had a follow-up of 60 months. Dissection of axillary lymph nodes was carried out in all of cases. None of them received radiotherapy, hormonal therapy or chemotherapy before surgery. After immunohistochemistry and Western blot analysis, we reviewed clinical records and identified two patients' groups: Group 1) Forty five patients did not relapse after a minimum period of 24 mo. of follow-up (follow-up median 57 months, range 24 to 61 mo.). Twenty four of the 45 cases showed no evidence of ganglionar lesions at diagnosis (53.3%) and 21 showed ganglionar metastasis (46.7%). Group 2) Thirty two patients who relapsed with a median disease free interval of 18.5 months (range 7 to 64 mo.). Three of these 32 cases showed no ganglionar lesions at diagnosis (9.4%) and 29 patients showed ganglionar metastasis (90.6%). In the group 1, nineteen patients received adjuvant therapy with tamoxifen, 20 were treated with chemotherapy and tamoxifen, 5 with chemotherapy only and 1 of them received radiotherapy. In the group 2, three patients received adjuvance with tamoxifen, 19 tamoxifen and chemotherapy, 4 received chemotherapy only and 5 adjuvant endocrine therapy without tamoxifen. 27 patients received a second-line of chemotherapy and 14 died between 2 and 32 months after the diagnosis of metastasis.

### Immunoblotting

For Western blot analysis, each sample was homogenised in 0.5 M Tris-HCl buffer (pH 7.4) containing 1 mM EDTA, 12 mM 2-mercaptoethanol, 1 mM benzamidine, and 1 mM phenylmethylsulphonyl fluoride (PMSF), with the addition of a cocktail of protease inhibitors (10 mM iodoacetamide, 0.01 mg/ml of soybean trypsin inhibitor and 1 μl/ml of leupeptin) and phosphatase inhibitors (10 mM sodium fluoride and 1 mM sodium orthovanadate) in the presence of 0.5% Triton X-100. Homogenates were centrifuged for 10 min at 15000 × g. After boiling for 2 min at 98°C, aliquots of 70 μg of protein were separated in SDS-polyacrylamide (9% w/v) slab minigels. Separated proteins were transferred for 4 h at 0.25 A to nitrocellulose membranes (0.2 μm) and, thereafter, the nitrocellulose sheets were blocked for 1 h with 5% blotto in 0.05 M Tris-HCl and incubated overnight with the primary antibodies diluted 1:200 (RXR-α and -γ), and 1:100 (RXR-β, hPXR1, and hPXR1.2) in blocking solution 1:9 overnight at 37°C.

For RXRs, the blots were incubated with peroxidase-linked secondary antibody (Chemicon) diluted 1:4000 for 1 hour at room temperature. For hPXR1 and hPXR1.2, swine anti-goat and goat anti-rabbit biotinylated immunoglobulins (Dako, Barcelona, Spain) were used at 1:1000 dilution in blocking solution 1:9 for 1 h at room temperature, and then the membranes were incubated with streptavidin-peroxidase complex (Zymed, CA, USA). Antibody/protein complexes were detected using ECL (Amersham, Buckinghamshire, UK).

Extracts from breast cancer cell lines (MCF-7 and MDA-MB-231) were used as positive controls for hPXR1.2 and hPXR1 antibodies. Blots were stripped and re-probed with an anti-human β-actin monoclonal antibody (Sigma) to control for equal sample loading.

### Immunohistochemistry

Sections of 5-μm-thickness were deparaffined, hydrated and incubated for 20 min in 0.3% H_2_O_2 _to inhibit endogenous peroxidase activity, and for antigen retrieval, incubated with 0.1 M citrate buffer (pH 6) for 10 min. at 96°C. After rinsing in TBS, the slides were incubated with 3% normal donkey serum (NDS) in TBS for 30 min to prevent non-specific binding of the first antibody. Afterwards, they were incubated overnight at 37°C with the RXR-α and RXR-γ rabbit polyclonal antibodies and RXR-β mouse monoclonal primary antibody (Santa Cruz Biotechnologies, CA, USA), diluted 1:20 in blocking solution 1:9; rabbit polyclonal hPXR (that reacts with the isoforms 1 and 2 of PXR) (Active Motif, Rixensart, Belgium) at 1:300 dilution, and goat polyclonal hPXR1 (isoform 1) diluted 1/20 (Santa Cruz). The sections were washed in TBS and incubated with swine anti-goat (for hPXR1), swine anti-rabbit (for hPXR1.2, RXR-α and RXR-γ), or rabbit anti-mouse (for RXR-β) biotinylated immunoglobulins (Dako, Barcelona, Spain) all of them at 1:400 dilution during 1 h. Thereafter, they were incubated with avidin-biotin-peroxidase complex (Dako) and developed with 3, 3'-diaminobenzidine (DAB) using the glucose oxidase-DAB-nickel intensification method. The sections were dehydrated, cleared in xylene, and mounted in DePex (Probus, Badalona, Spain).

To assess the specificity of immunoreactions, negative and positive controls were used. As negative controls, sections of breast samples processed identically were incubated using the antibody preabsorbed with corresponding blocking peptide, or omitting the primary antibody. As positive controls, sections of human liver, intestine for hPXR1.2 and hPXR1, and human skin for the three isoforms of RXR were processed with the same antibody.

The staining intensity of hPXR and RXRs receptors was classified in two categories: 0, negative or staining was observed in less than 10% of the cells; 1, staining was detected in more than 10% of the cells. In contrast to nuclear staining, the staining pattern of the extranuclear expression for these proteins was observed in two types: diffuse staining and spotted staining in the cytoplasm according to the following criteria: score 0, no staining at all; 1, a weak staining; 2, a moderate to strong staining was observed in more than 10% of the tumour cells. The assessment of the grade of staining was performed in a blinded way always by the same experienced investigators (IC, MIA) in high-power fields (×400) using standard light microscopy.

### Statistical analysis

To evaluate the differences between hPXR and RXRs expression for each of the different pathology types (BBDs, CIS, IDC and ILC), we performed overall comparisons using non-parametric ANOVA (Kruskal-Wallis test). In infiltrative carcinomas, univariate analysis comparing categorical variables (hPXR, RXR, ER and PR expression and clinicopathological data) was performed using chi-square tests. Given the low expected frequencies found in the majority of the crosstabulations, we used Fisher exact test to compute *p*-values. We test for the presence of a linear trend when there were more than two categories of staining using Mantel-Haenszel chi-square statistic. Time to post-operative recurrence was analyzed using Kaplan-Meier estimations of disease free survival curves. Survival curves were compared with log-rank test. We adopt a 5% significance level. All analyses were performed with SPSS version 13.0 for Windows.

## Results

### Western blot analysis

**Figure 1:**
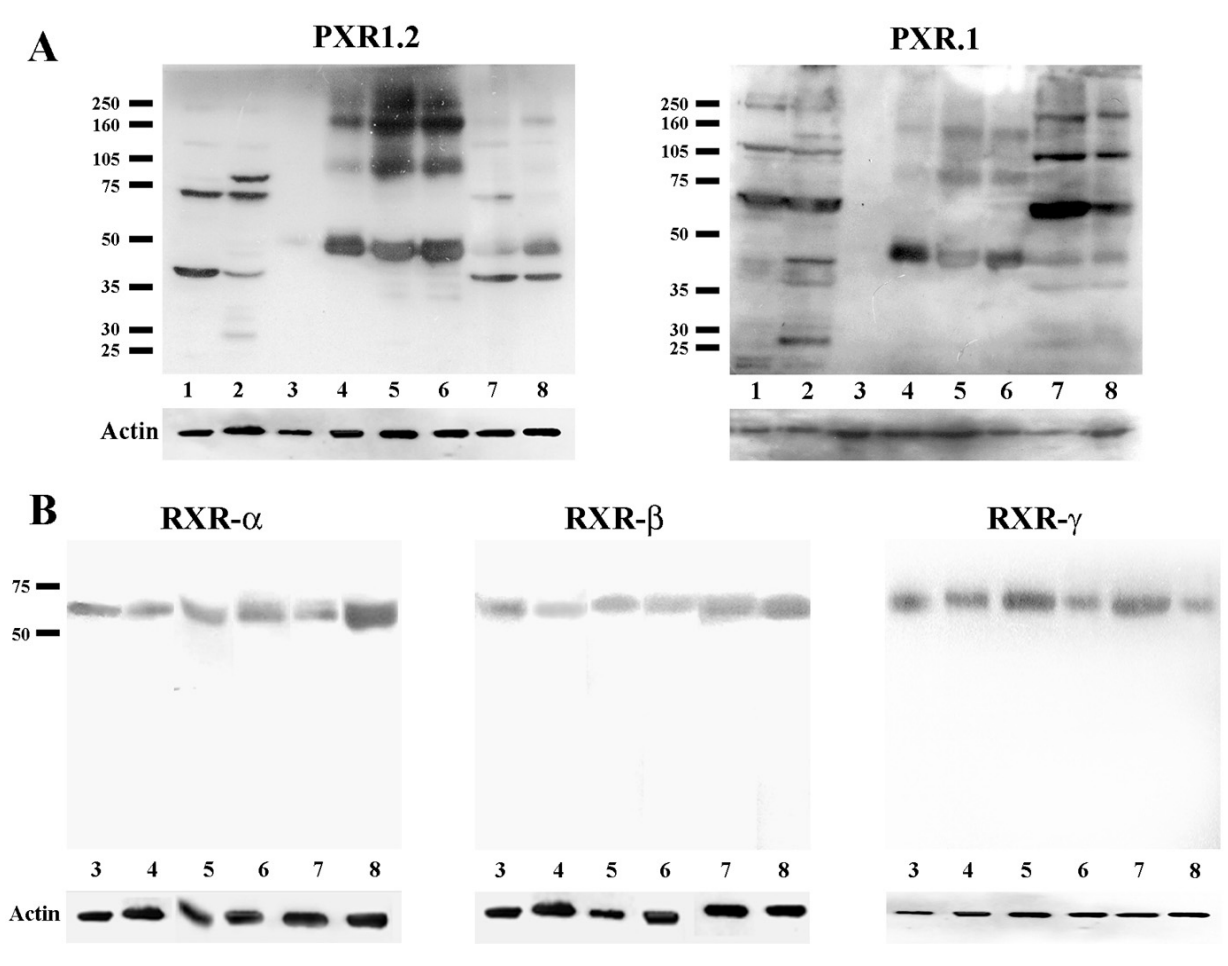
**A. Western blot analysis for hPXR1.2 and hPXR1 antibodies**. With hPXR1.2 antibody, in MCF-7 cells were detected mainly two bands at 40 and 70 kDa; however, in MDA-MB-231 can be also observed additional bands at 28 and 90 kDa. In this last cell line, with hPXR1 antibody multiple bands at 28, 37, 40, 70, 120 and 250 kDa; however, in MCF-7 were only detected bands at 70, 120 and 250 kDa. In benign breast diseases no bands were observed either with hPXR1.2 or with hPXR1 antibody. In carcinomas *in situ*, bands at 50, 100 and 160 kDa were observed with both antibodies. In infiltrative carcinomas, those samples incubated with hPXR1.2 presented multiple bands at approximately 40, 50 and 70 kDa.; while in samples incubated with hPXR1 antibody, five immunoreactive bands at 40, 50, 70, 120 and 250 kDa were observed. **B. Western blot analysis for RXRs antibodies**. For RXR-α, RXR-β and RXR-γ, only a single band at 60 kDa of molecular weight was found in all pathologies studied. For all figures: Lane 1: MCF-7 cells. Lane 2: MDA-MB-231 cells. Lane 3: benign breast diseases. Lane 4: Ductal carcinoma *in situ*. Lanes 5 and 6: Lobular carcinoma *in situ*. Lane 7: Infiltrative ductal carcinoma. Lane 8: Infiltrative lobular carcinoma. Each blot is representative of its respective group. After stripping, immunoreactivity with an anti-actin antibody was used as loading control (actin, bottom panels).

Results from Western blot analysis are shown in Figure 1. In breast cancer cell lines, hPXR1.2 antibody showed two distinctive immunoreactive bands at 40 and 70 kDa; in MDA-MB-231 cells an additional band at 90 kDa and other fainter band at ~28 kDa were observed. In CIS, three bands were detected at 50, 100 and 160 kDa. Infiltrative carcinomas showed a strong band at 40 kDa, and additional protein bands at 50 and 70 kDa. When the same samples were incubated with the antibody that exclusively recognizes the hPXR1 isoform, multiple bands were observed in MDA-MB-231 cells, at approximately 28, 40, 70, 120 and 250 kDa (Fig. 1B). In MCF-7 cells only the 70, 120 and 250 KDa were detected. Carcinomas *in situ *samples showed the same three bands detected with hPXR1.2 antibody at 50, 100 and 160 kDa. In infiltrative carcinomas, hPXR1 antibody detected immunoreactive protein bands at approximately 50, 70, 120 and 250 kDa. No immunoreaction to hPXR1.2 or to hPXR1 was detected in samples from benign breast diseases.

In all the samples studied, RXR-α, RXR-β and RXR-γ antibodies showed a single band with a molecular weight of 60 kDa.

### Immunohistochemical study of control sections

**Figure 2:**
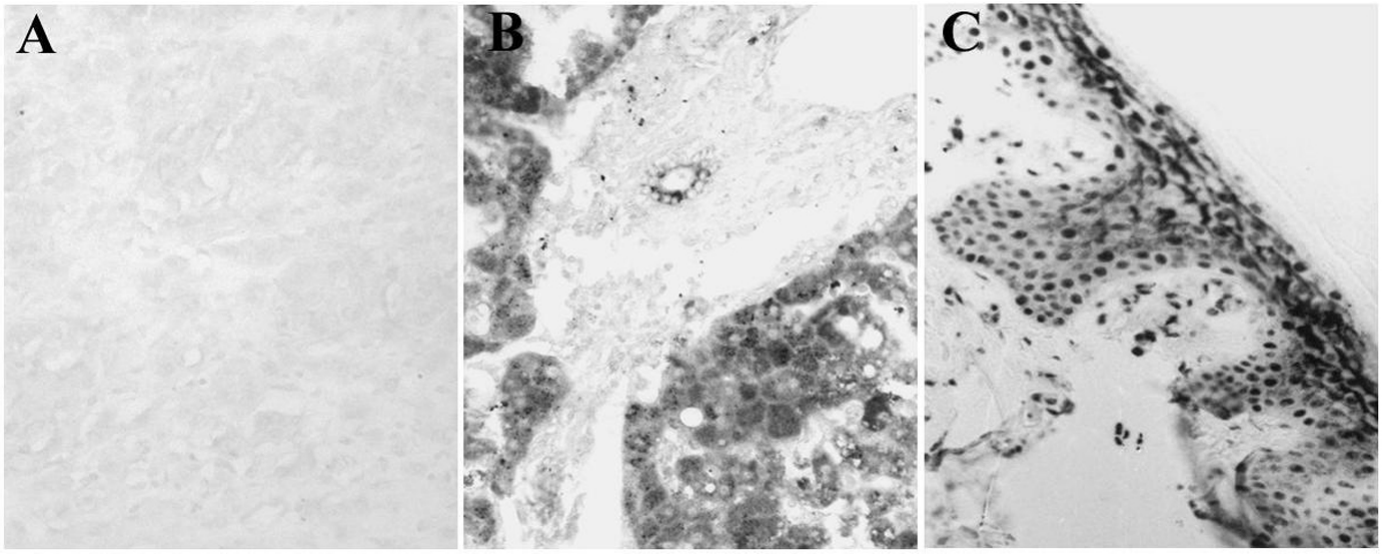
**A**. Negative control section of infiltrative ductal carcinoma was obtained when it was incubated with antibody pre-absorbed with blocking peptide (×250).**B**. Control section from human liver with an intense reaction to hPXR1.2 antibody in the cytoplasm of hepatocytes (×250). **C**. Control section of human skin. The nuclei of keratinocytes were intensely labelled for RXRa (×250).

The immunohistochemical study showed no reaction in the negative controls obtained when they were incubated with antibody pre-absorbed with blocking peptide (Fig. 2A). Positive controls for hPXRs antibodies showed an intense immunoreaction in the cytoplasm of hepatocytes (Figure 2B). Immunostaining of human skin sections were always positive to RXRs antibodies (Figure 2C).

### Immunohistochemical detection of hPXR

**Figure 3:**
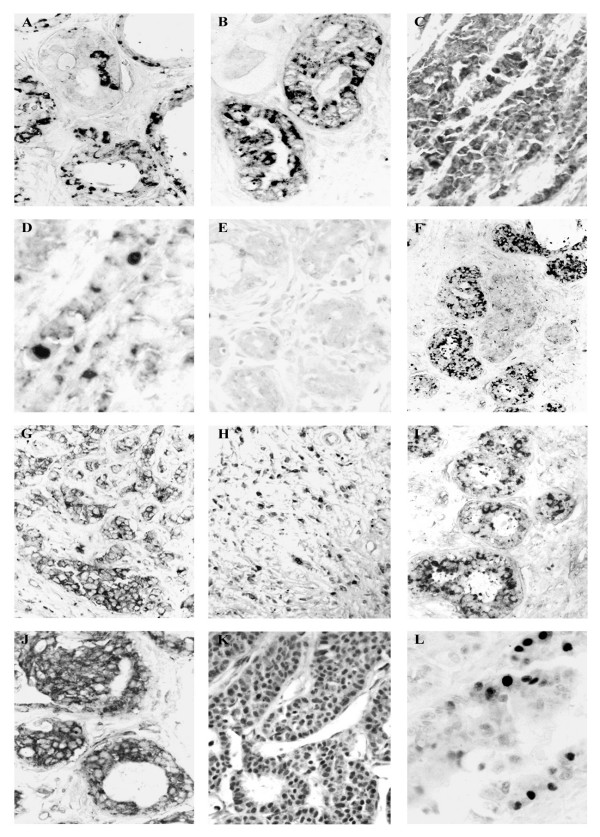
**Immunohistochemical detection of hPXR and RXR receptors**. **Immunoreaction to hPXR1.2 (A, B, C and D)**. In ductal hyperplasia (**A**) (×250) and ductal carcinoma *in situ *(**B**) (×250), the reaction was observed in the cytoplasm. In infiltrative ductal carcinoma (**C**) (×250) and infiltrative lobular carcinoma (**D**) (×500), the hPXR1.2 immunoexpression was also observed in the nucleus of neoplastic cells. **Immunoreaction to hPXR1 (E, F, G and H)**. Lobular hyperplasia (**E**) (×200) showing no immunoreaction to hPXR1 antibody. Micrograph of lobular carcinoma *in situ *(**F**) (×200), infiltrative ductal carcinoma (**G**) (×200) and infiltrative lobular carcinoma (**H**) (×200) with cytoplasmic immunolocation of hPXR1 isoform. **Immunoreaction to RXR-α (I, J, K and L)**. Cytoplasmic immunolabelling of RXR-α in samples of ductal hyperplasia (**I**) (×200) and ductal carcinoma *in situ *(**J**) (×300). Neoplastic cells from infiltrative ductal carcinoma (**K**) (×300) and infiltrative lobular carcinoma (**L**) (×500) presenting a nuclear immunoexpression to RXR-α.

hPXR1.2 was detected in the cytoplasm in the most of samples (Table 1). In normal breast ducts and acini, only some myoepithelial and endothelial cells were immunoreactive. In BBDs, a cytoplasmic immunoreaction to hPXR1.2 in the epithelial cells was also detected (Fig. 3A). Carcinomatous lesions showed some variations in hPXR1.2 expression; thus, 90% of CIS showed cytoplasmic reaction (Fig. 3B); while in IDC, the 100% of samples presented cytoplasmic reaction, and the 45.8% of samples showed nuclear immunolabelling (Fig. 3C). Infiltrative lobular carcinomas showed higher nuclear immunoreaction (80%) (Fig. 3D).

**Table 1:** Immunohistochemical expression of Retinoid X receptors and hPXR isoforms in human breast lesions.

	**BBDs (n = 12)**		**CIS (n = 10)**		**IDC (n = 54)**		**ILC (n = 23)**		***p*-value**^1^	
	
	**N**	**C**	**N**	**C**	**N**	**C**	**N**	**C**	**N**	**C**
**hPXR1.2**	0	6 (50%)	0	9 (90%)	15 (27.8%)	54 (100%)	7 (30.4%)	23 (100%)	0.048	**<0.001**
**hPXR1**	0	4 (33.4%)	0	8 (80%)	0	54 (100%)	0	23 (100%)	1.000	**<0.001**
**RXR-**α	0	1 (8.4%)	0	2 (20%)	23 (42.6%)	25 (46.3%)	11 (47.8%)	8 (34.8%)	**0.002**	**0.037**
**RXR-**β	0	0	0	3 (30%)	8 (14.8%)	30 (55.6%)	2 (8.7%)	12 (52.1%)	0.301	**0.003**
**RXR-**γ	0	2 (16.7%)	0	4 (40%)	8 (14.8%)	35 (64.8%)	5 (21.7%)	10 (43.5%)	0.161	**0.002**

Numbers represent the frequencies and percentages of cases with positive reaction. **BBDs**: benign breast diseases. **CIS**: carcinoma *in situ*. **IDC**: infiltrative ductal carcinoma. **ILC**: infiltrative lobular carcinoma. **n**: number of cases. **N**: nucleus. **C**: cytoplasm. ^1^*p*-values obtained by one-way Kruskal-Wallis test to compare overall between pathological groups differences in the degree of staining.

hPXR1 isoform showed a similar expression pattern to hPXR1.2 in benign lesions and CIS (Figs. 3E and 3F). However, infiltrative carcinomas only showed cytoplasmic immunoreactivity (Figs. 3G and 3H).

The most important difference between the patient groups was that patients who evolved to recurrent disease showed cells with nuclear staining to PXR1.2 antibody, but these nuclei were consistently immunonegative to PXR.1.

### Immunohistochemical expression of retinoid receptors

Both benign samples and CIS showed only cytoplasmic immunoreaction to RXR-α (Figs. 3I and 3J). However, in both ductal and lobular infiltrative carcinomas this receptor was also observed in the nucleus of neoplastic cells (Figs. 3K and 3L).

The percentage of positive cases for RXR-β was similar to that of RXR-α in BBDs and CIS, and lower in infiltrative carcinomas; in IDC, only one case of nuclear immunoreaction was observed.

The immunoexpression observed for RXR-γ was similar to that of RXR-α, although the percentage of samples with cytoplasmic immunoreaction was always higher and lower that of nuclear expression.

### Statistical analysis

The Fisher's exact tests realized between the RXR and hPXR isoforms (Table 2) showed a positive association between the expression of hPXR isoforms and nuclear and cytoplasmic RXR-α expression. Also, a positive association between the expression of both hPXR isoforms and cytoplasmic expression of both RXR-β and RXR-γ was encountered.

**Table 2:** Fisher's exact test between the expression of the different isoforms of hPXR and that the retinoid receptors in infiltrative carcinomas.

			**hPXR1.2 C**			**hPXR1.2 N**			**hPXR1 C**		
		
		**Total**	**Weak**	**High**	**p-value**	**Negative**	**Positive**	**p-value**	**Weak**	**High**	**p-value**
**RXR-α C**	**Negative**	45	17	28	**0.035**	24	21	**<0.001**	19	26	**0.007**
	**Weak**	6	5	1		6	0		5	1	
	**High**	26	16	10		25	1		20	6	
	**Total**	77	38	39		55	22		44	33	
											
**RXR-α N**	**Negative**	43	30	13	**<0.001**	42	1	**<0.001**	34	9	**<0.001**
	**Positive**	34	8	26		13	21		10	24	
	**Total**	77	38	39		55	22		44	33	
											
**RXR-β C**	**Negative**	35	18	17	**0.021**	22	13	**0.045**	18	17	0.329
	**Weak**	18	13	5		17	1		13	5	
	**High**	24	7	17		16	8		13	11	
	**Total**	77	38	39		55	22		44	33	
											
**RXR-β N**	**Negative**	67	32	35	0.470	48	19	0.915	38	29	0.845
	**Positive**	10	6	4		7	3		6	4	
	**Total**	77	38	39		55	22		44	33	
											
**RXR-γ C**	**Negative**	32	21	11	**0.001**	28	4	**0.001**	24	8	**<0.001**
	**Weak**	12	9	3		11	1		10	2	
	**High**	33	8	25		16	17		10	23	
	**Total**	77	38	39		55	22		44	33	
											
**RXR-γ N**	**Negative**	64	34	30	0.142	46	18	0.847	37	27	0.792
	**Positive**	13	4	9		9	4		7	6	
	**Total**	77	38	39		55	22		44	33	

**C**: Cytoplasmic immunoreaction. **N**: nuclear immunoreaction.

**Figure 4:**
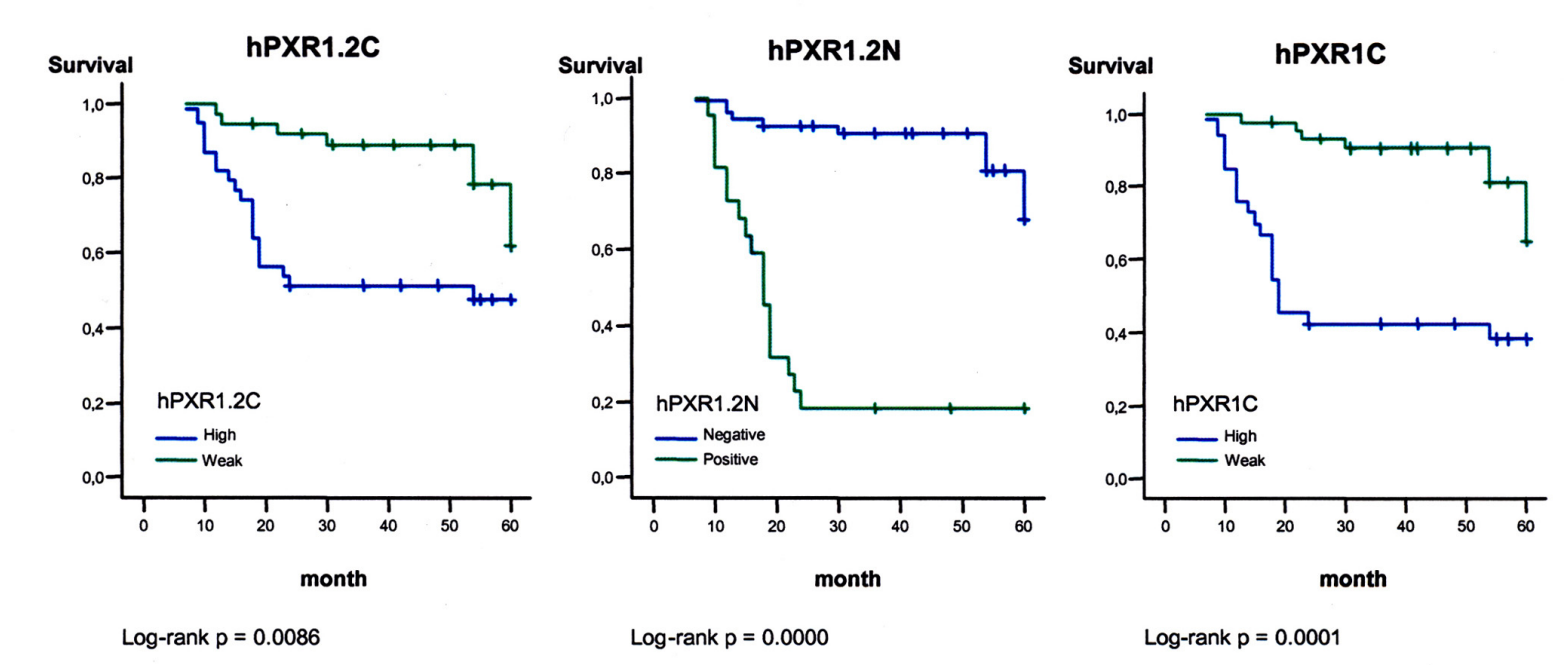
**Kaplan-Meier disease-free survival curves for 77 patients with infiltrative carcinomas according to the hPXR expression.** Marks represent censored data. Statistical significance was determined by the Log Rank test.

The associations between the hPXR isoforms expression and the clinicopathological data of patients are reflected in Table 3. Patient's age was homogeneous and independent on hPXR results. hPXR expression was not associated with tumour type. The nuclear hPXR1.2 expression was significantly more frequent in patients with positive nodal status. hPXR expression was inversely correlated with ER expression while that of PR was correlated with the nuclear and cytoplasmic expression of hPXR1.2 and the hPXR1 cytoplasmic expression. hPXR expression was inversely correlated with recurrence and disease-free interval (Fig. 4).

**Table 3:** Fisher's exact test between hPXR expression and different clinicopathological data of the patients presenting infiltrative carcinomas.

			**hPXR1.2 C**			**hPXR1.2 N**			**hPXR1 C**		
		
		**Total**	**Weak**	**High**	**p-value**	**Negative**	**Positive**	**p-value**	**Weak**	**High**	**p-value**
**Tumor Type**	**Ductal**	54	26	28	0.613	39	15	**0.002**	33	21	0.374
	**Lobular**	22	12	10		16	6		11	11	
											
**Age**	**<50**	19	13	6	0.055	13	6	0.738	12	7	0.542
	**>50**	58	25	33		42	16		32	26	
											
**Tumor size**	**1**	31	20	11	0.083	27	4	0.077	23	8	0.088
	**2**	33	15	18		21	12		16	17	
	**3**	5	1	4		3	2		2	3	
	**4**	8	2	6		4	4		3	5	
											
**Nodal status**	**-**	35	20	15	0.212	30	5	**0.011**	21	14	0.644
	**+**	42	18	24		25	17		23	19	
											
**ER**	**0**	17	7	10	0.257	9	8	**0.001**	10	7	0.161
	**1**	6	1	5		1	5		1	5	
	**2**	12	6	6		9	3		6	6	
	**3**	42	24	18		36	6		27	15	
											
**PR**	**0**	21	10	11	**0.031**	14	7	**0.000**	12	9	**0.004**
	**1**	7	0	7		0	7		0	7	
	**2**	13	6	7		10	3		6	7	
	**3**	36	22	14		31	5		26	10	

**Tumor size (TNM)**: T = 1, 2, 3, 4. **ER **and **PR**: **0 **= Negative, **1 **= Weak immunoreaction, **2/3 **= High immunoreaction. **C**: Cytoplasmic immunoreaction. **N**: nuclear immunoreaction.

**Figure 5:**
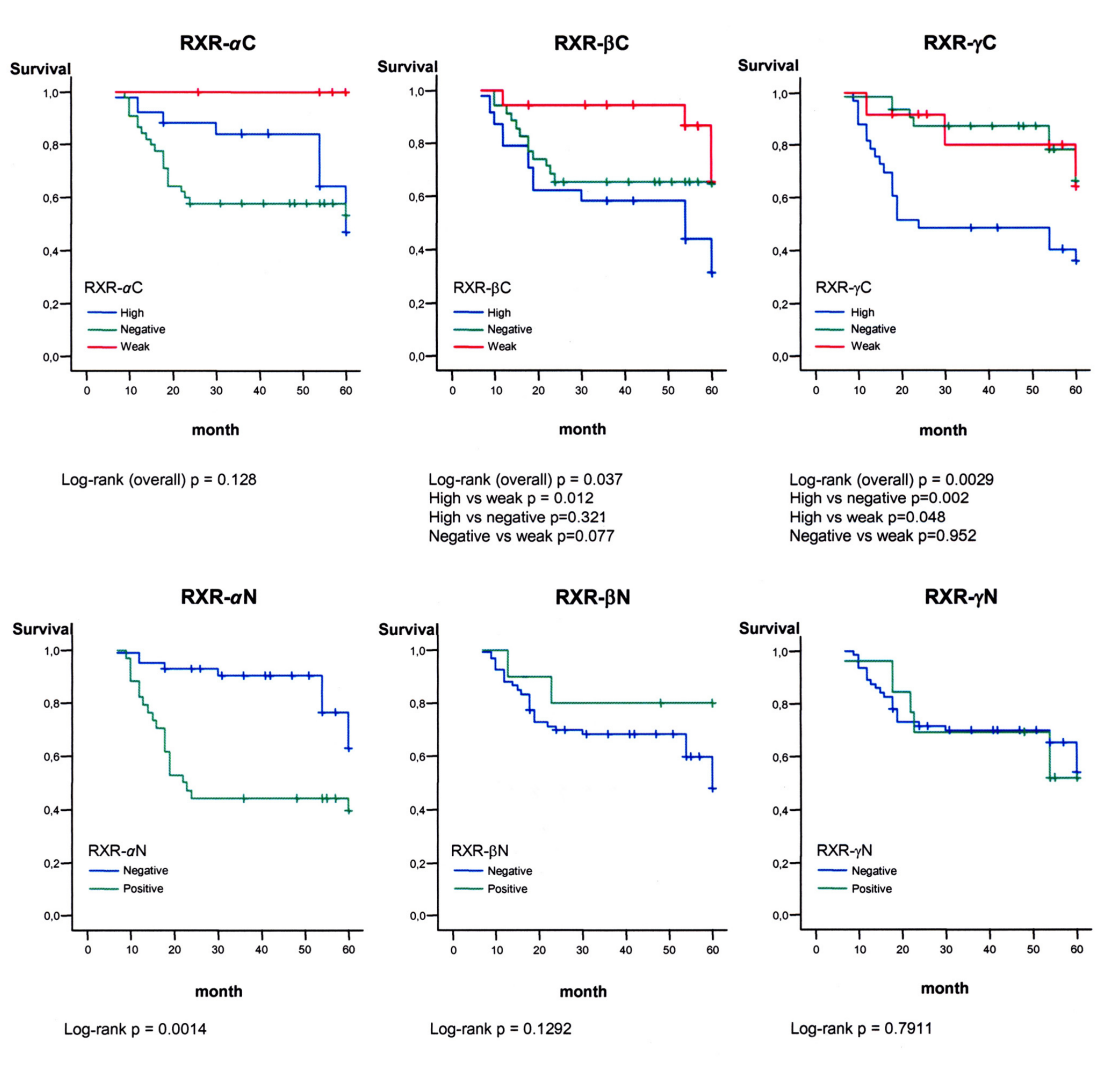
**Kaplan-Meier disease-free survival curves for 77 patients with infiltrative carcinomas according to the RXR expression.** Marks represent censored data. Statistical significance was determined by the Log Rank test.

The associations between the RXRs expression and the clinicopathological data are reflected in Tables 4 and 5. Nodal status was correlated with cytoplasmic expression of both RXR-β and RXR-γ. The cytoplasmic expression of RXR-γ was inversely correlated with ER and PR expression and RXR-α nuclear expression was correlated with that of PR. Relapse and survival were correlated with cytoplasmic expression of RXR-β and RXR-γ and with nuclear expression of RXR-α (Fig. 5).

**Table 4:** Fisher's exact test between RXRs cytoplasmic expression and different clinicopathological data of the patients presenting infiltrative carcinomas.

		**RXR-α C**				**RXR-β C**				**RXR-γ C**			
		
		**Negative**	**Weak**	**High**	**p-value**	**Negative**	**Weak**	**High**	**p-value**	**Negative**	**Weak**	**High**	**p-value**
**Tumor Type**	**Ductal**	30	3	21	0.264	24	12	18	0.838	19	10	25	0.151
	**Lobular**	14	3	5		10	6	6		13	2	7	
													
**Age**	**<50**	12	3	4	0.185	10	4	5	0.765	5	3	11	0.254
	**>50**	33	3	22		25	14	19		27	9	22	
													
**Tumor size**	**1**	15	4	12	0.697	12	11	8	0.292	15	7	9	0.392
	**2**	22	2	9		17	4	12		12	5	16	
	**3**	3	0	2		1	2	2		2	0	3	
	**4**	5	0	3		5	1	2		3	0	5	
													
**Nodal status**	**-**	22	3	10	0.678	17	12	6	**0.024**	18	9	8	**0.003**
	**+**	23	3	16		18	6	18		14	3	25	
													
**ER**	**0**	10	0	7	0.274	6	2	9	0.060	2	3	12	**0.000**
	**1**	6	0	0		3	0	3		1	0	5	
	**2**	6	2	4		4	6	2		2	4	6	
	**3**	23	4	15		22	10	10		27	5	10	
													
**PR**	**0**	13	1	7	0.296	11	5	5	0.480	9	2	10	**0.009**
	**1**	7	0	0		3	0	4		0	0	7	
	**2**	8	1	4		5	5	3		4	5	4	
	**3**	17	4	15		16	8	12		19	5	12	

**Tumor size (TNM)**: T = 1, 2, 3, 4. **ER **and **PR**: **0 **= Negative, **1 **= Weak immunoreaction, **2/3 **= High immunoreaction. **C**: Cytoplasmic immunoreaction.

**Table 5:** Fisher's exact test between RXRs nuclear expression and different clinicopathological data of the patients presenting infiltrative carcinomas.

		**RXR-α N**			**RXR-β N**			**RXR-γ N**		
		
		**Negative**	**Positive**	**p-value**	**Negative**	**Positive**	**p-value**	**Negative**	**Positive**	**p-value**
**Tumor Type**	**Ductal**	31	23	0.819	46	8	0.503	46	8	0.406
	**Lobular**	12	10		20	2		17	5	
										
**Age**	**<50**	9	10	0.391	17	2	0.713	17	2	0.394
	**>50**	34	24		50	8		47	11	
										
**Tumor size**	**1**	21	10	0.304	27	4	0.829	26	5	0.123
	**2**	17	16		28	5		30	3	
	**3**	2	3		5	0		3	2	
	**4**	3	5		7	1		5	3	
										
**Nodal status**	**-**	19	16	0.802	28	7	0.095	28	7	0.505
	**+**	24	18		39	3		36	6	
										
**ER**	**0**	8	9	0.093	15	2	0.254	16	1	0.116
	**1**	1	5		6	0		6	0	
	**2**	6	6		12	0		11	1	
	**3**	28	14		34	8		31	11	
										
**PR**	**0**	13	8	**0.010**	18	3	0.608	17	4	0.641
	**1**	0	7		7	0		7	0	
	**2**	6	7		12	1		11	2	
	**3**	24	12		30	6		29	7	

**Tumor size (TNM)**: T = 1, 2, 3, 4. **ER **and **PR**: **0 **= Negative, **1 **= Weak immunoreaction, **2/3 **= High immunoreaction. **N**: nuclear immunoreaction.

## Discussion

hPXR has been shown to activate transcription of reporter genes through a response element conserved in the promoter of the *CYP3A *genes [3,5], suggesting that hPXR might be a transcriptional regulator of CYP3A expression [5]. Because these CYP3A enzymes have also been found in human breast cancer tissues [13,18], hPXR/CYP3A-regulated pathways might be involved in therapy response of breast cancer. Thus, the first step to evaluate the functions of hPXR/CYP3A in breast cancer it would be to determinate the expression pattern of the hPXR proteins. This study provides evidence that both hPXR isoforms are expressed in human breast cancer but not in normal glands, although a previous report showed that theirs transcripts are expressed in both normal and neoplastic human breast tissue [14]. Our findings suggest that translation of mRNA might only occurs in breast cancer; similar results have been reported by Miki et al. [15] who detected the presence of hPXR mRNA and protein in breast carcinomatous tissues but not in nonneoplastic and stromal cells.

The presence of different hPXR isoforms in breast lesions has been observed by Western blot analysis. By using MCF-7 and MDA-MB-237 cells to control antibodies immunoreactivity, we observed that the latter presented multiple distinctive bands and that the bands at 28 and 90 kDa probably belong to the hPXR.2 isoform. Samples from CIS showed identical immunoreactive bands with the two antibodies we used. Furthermore, infiltrative carcinomas showed a similar pattern to that encountered in MDA-MB-231 cells. These results agree with previous findings that suggest that hPXR is expressed as multiple forms, due to alternative as well as defective gene splicing [19]; therefore, in the same tissue, interindividual differences in hPXR transcript and protein profiles may exist. In addition, a different expression of three PXR isoforms have been detected in human liver and hepatoma cells, stomach, adrenal gland, bone marrow and brain [2,20].

A previous report showed that mRNA of hPXR variants 1 and 2 is expressed in several cell lines, such as MCF-7 or MDA-MB-231 [14], and this expression is inversely related to the ER status. The ER status is used as a therapeutic indicator and is also a prognostic marker in human breast cancer [21]. We have observed that the neoplastic cells that express nuclear hPXR1.2 and no ER immunoreactivity were strongly correlated; this inverse correlation between both receptors suggests that pathways by hPXR-mediated might be functional in breast tumours with potential poor response to the endocrine adjuvance. This correlation between ER and hPXR was also found by Masuyama et al. [23] in human endometrium; these authors only detected hPXR expression in endometrial cancer tissues but not in normal endometrium and, similarly they encountered a significant inverse correlation between the expression of PXR and ER. Altogether, these results suggest that hPXR might play some role in the metabolism of steroid hormones in tumoral cells, and that may be involved in the growth and development of cancer tissue that express low ER-alpha.

We have also analysed ER and hPXR cytoplasmic expression observing the same inverse correlation between both receptors as that encountered for the nuclear immunolocation. This is in agreement to the observation by Dotzlaw et al. [14] who reported that MCF-7 cells (ER+/PR+), with a low metastatic potential, express hPXR1 mRNA but not hPXR.2 mRNA. However, MDA-MB-231 cells (ER-/PR-) with a high metastatic potential, showed the highest levels of both hPXR1 and 2 mRNA variants.

In breast cancer, the hPXR isoform 2 seems to be a functional protein since it was detected in the cell nuclei. It has been reported, in mouse liver, that PXR is retained in the cytoplasm in a complex formed by hsp90 and the co-chaperone CCRP, and in presence of ligand, PXR is accumulated in the nucleus [24]. We have observed an increase of the hPXR expression in both nucleus and cytoplasm related to breast cancer progression; the biological significance of this rise correlated to neoplastic transformation is unknown; clearly, more studies are needed to elucidate this accumulation. Although the percentage of samples with nuclear immunostaining was lower than the cytoplasmic staining, the specimens with nuclear reaction corresponded to all those cases that presented resistance to conventional treatments and that metastasized later. Therefore, an important correlation between cancer recurrence and nuclear immunostaining of hPXR1.2 was observed.

RXRs are a member of the larger steroid/thyroid receptor superfamily where all members are ligand-activated transcription factors, among them RXR is unique to this group for its ability to interact with other receptors and form heterodimers complexes [25,26]. For Lawrence et al. [27], the overexpression of RXRs isoforms in ductal carcinoma *in situ*, especially RXR-α, indicate an association with an increased risk for the development of invasive breast cancer. Ariga et al. [28] detected a widely distributed expression of the three RXR isoforms in ductal carcinomas *in situ*; however, other authors detected RXR-γ expression neither in breast cancer cell lines [28] nor in invasive ductal breast carcinoma [29]. None of these authors have reported cytoplasmic expression for these receptors that we have detected, the differences in immunolocation might be related to either the absence of ligand or the antibody used. Moreover, it has been shown that some nuclear receptors (steroids receptors) are found as an inactive cytoplasmic form in a complex with heat shock proteins [30]. It is also possible that inactive retinoic acid nuclear receptors were forming a complex with heat shock proteins in the cytoplasm. With breast cancer progression, we have detected an increase in the percentage of samples positives for RXRs isoform; since RXR are needed partners of different nuclear receptors and there is an alternative pathway of activation for RXR as a homodimer by binding to a unique response element [31], this increase could be related to the several modifications that occur in the malignant progression.

The function of each RXR subtype in the mammary gland has yet to be defined although some studies have demonstrated an interaction between estrogen action and RXR specifically with RXR-α [29,32]. The correlation between the expression of RXR-α and hPXR observed in this study supports the idea of dimerization of both receptors. Previous reports showed that different xeno-sensor target genes have different sensitivity to different xenobiotics and that RXR-α has an effect in gene regulation whereas RXR-β and RXR-γ do not [4]. Cai et al. [22] demonstrated similar data in mice that carried a RXR-α mutation; in hepatocytes, the level of RXR-α controls the basal transcription of *CYP450 *genes.

One of the most important drugs developed for breast cancer treatment is tamoxifen, used for systemic treatment for nearly three decades [33,34]. Although tamoxifen has been shown to be effective in the most tumours, some of them are unresponsive, by acquiring eventually resistance to this drug or well their growth becomes stimulated by it. There are many possible mechanisms to explain this resistance including the down-regulation, mutation, or loss of estrogen receptors, impaired co-activator signalling, and altered tamoxifen pharmacology [35].

The metabolism of tamoxifen is mainly regulated by the P450 family of cytochromes, which catalyze its conversion to both active and inactive products. In human liver CYP3A4 regulates the conversion of tamoxifen into its main active metabolite, 4-OH-tamoxifen [36]. Also, hepatic CYP3A4 can be up-regulated by hPXR throughout a mechanism involving the tamoxifen/4-OH-tamoxifen [37].

In normal human breast and breast cancer several CYP450 enzymes has been described [16,18,38], as well as the expression of the CYP3A mRNA splicing forms [18]. Also, these studies showed a possible relationship among the different enzyme subclasses and the development, progression or response to antineoplastic agents [39]. Therefore, the machinery for possible *in situ *bioactivation of xenobiotics and modification of therapeutic drugs is present in human breast tissue. There are few available data about the relationship between tamoxifen and hPXR/CYP450, Sane et al. [40] have reported that the CYP3A4 induction by tamoxifen and 4-OH-tamoxifen is primarily mediated by hPXR but the overall stoichiometry of other nuclear receptors such as GR and ER-α also contribute to the extent of the inductive effect. Huang et al. [18] considered that intratumoral hPXR levels might be useful as a predictor of response to adjuvant therapy in breast cancer patients. Synold et al. [41] have proposed that a classification of tumours as hPXR-positive or hPXR-negative might help predict whether the tumour is likely to develop chemotherapy-induced drug resistance. Our results agree with those observations since we observed that the higher levels of hPXR variants 1 and 2 are related to shorter disease-free intervals and either local recurrences or progressive disease.

## Conclusion

In infiltrative carcinomas, the isoform responsible for hPXR activity is the isoform 2. This isoform binds especially to RXR-α to form heterodimers which that activate transcriptional pathways in breast neoplastic cells. Since nuclear immunolocation occurs in samples from patients who presented recurrence, we suggest that the overexpression and the subcellular location changes of hPXR could be considered as a potential new prognostic indicator.

## Competing interests

The authors declare that they have no competing interests.

## Authors' contributions

IC and MIA designed the study, carried out the immunohistochemistry studies and have been involving in drafting the manuscript. MVTL and RP participated in Western blot analysis, result interpretation and discussion. JP participated in the immunohistochemistry studies. FJG and EA prepared and provided the tumour biological samples, reviewed the patients' histories and participated in the immunohistochemistry studies. JZ performed the statistical analysis and participated in discussion. MIA and BF participated in study coordination and supervision. All authors read, discussed and approved the final manuscript.

## Pre-publication history

The pre-publication history for this paper can be accessed here:


